# The Effect of Charcot Neuroarthropathy on Limb Preservation in Diabetic Patients with Foot Wound and Critical Limb Ischemia after Balloon Angioplasty

**DOI:** 10.1155/2017/5670984

**Published:** 2017-08-29

**Authors:** Mehmet Burak Çildağ, Ömer Faruk Kutsi Köseoğlu

**Affiliations:** Department of Interventional Radiology, Adnan Menderes University Medicine Faculty, 09100 Aydın, Turkey

## Abstract

**Objective:**

The aim of this article is to investigate one-year limb preservation rates after below-the-knee angioplasty in patients with diabetic foot wound who only have critical limb ischemia (CLI) and those who have Charchot neuroarthropathy (CN) accompanied by CLI.

**Methods:**

This single-center, retrospective study consists of 63 patients with diabetic foot wound who had undergone lower extremity balloon angioplasty of at least 1 below-the-knee (BTK) vessel. Only those patients with postprocedural technical success of 100% were selected from the database. All patients were classified into two groups as patients with CLI and CN and patients with CLI only without CN. The Kaplan-Meier method was used to compare the limb preservation rates for the two groups.

**Results:**

There was no statistically significant difference between patient age, gender, diabetic disease duration, and comorbid disease such as chronic renal insufficiency, hypertension, and coronary artery disease of the two groups (*p* > 0.05). Limb preservation in the 12 months was 59.1% in the CN group and 92.7% in the group without CN. Also, limb preservation rates between the two groups displayed statistically significant differences (*p* < 0.005).

**Conclusion:**

This study showed that CLI can accompany CN in patients with diabetes. Limb preservation rates with endovascular treatment in diabetic patients with CLI only are better than in diabetic patients with CLI and CN.

## 1. Introduction

Peripheral artery disease (PAD) can be defined as a group of disorders characterized by narrowing and obstruction of the arteries that reduce blood flow. Critical limb ischemia (CLI) is a clinical condition characterized by PAD-related ischemic tissue loss including incurable ulcers or gangrene and ischemic pain at rest. Within 1 year of CLI diagnosis, 30% of cases undergo major amputation and 25–30% die [1]. Patients who develop CLI require early revascularization due to poor prognosis. CLI is common in diabetic patients and below-the-knee (BTK) arteries are usually affected [2]. In recent years, endovascular therapy has replaced surgical bypass treatment as a revascularization method especially in BTK arteries. Endovascular revascularization is now the first choice in CLI cases of BTK level [3, 4]. Also, Charcot neuroarthropathy (CN) is the other causative condition of limb loss in patients with diabetes. CN is a progressive disease involving the foot and ankle bones, joints, and soft tissues. It occurs in 0.1–7.5% of all patients with diabetes and 29% of diabetes patients with peripheral neuropathy [5, 6]. Major amputation rate increases especially in CN accompanied by ulcers or osteomyelitis [7]. Apart from a few studies, the literature has insufficient studies reporting PAD or CLI incidence in CN [8]. Due to the natural course of diabetes mellitus, PAD and CLI can be expected in CN cases. According to our clinical observations, the coexistence of CN and CLI is not so rare. We sometimes even have difficulty in differentiating whether it is due to ischemic or neuropathic origin when a diabetic patient with CN develops a foot wound and consider the case as neuroischemic. In addition, in cases with foot wound and CN accompanied by CLI, delayed wound healing after minor amputation and secondary infection may occur which ultimately may require major amputation. Although there are studies in the literature showing the effectiveness of endovascular treatment in cases with diabetic foot wound and CLI, there are no studies showing the effectiveness of endovascular treatment in cases with CN accompanied by CLI.

In this study, we investigated one-year limb preservation rates after angioplasty in patients with diabetic foot wound who only have CLI, and those who have CN accompanied by CLI.

## 2. Material and Method

### 2.1. Study Population and Design

This is a retrospective, single-center study based on the collected data of patients with diabetic wound who had undergone lower extremity percutaneous balloon angioplasty (PBA) of at least 1 BTK vessel. After institutional review board approval, imaging data between October 2014 and March 2016 were reviewed.

Inclusion criteria were the presence of diabetic foot wound and CLI, patients who had plain radiography of a damaged foot and patients who had undergone balloon angioplasty for stenosis or occlusion of at least 1 BTK vessel with distal runoff to the foot with technical success.

Exclusion criteria were planned major amputation before angiography and unsuccessful PTA, patients whose data constituted any intervention before angioplasty, who did not have plain radiography of the foot, who had suspicion of acute CN, and who had concomitant above-knee arterial steno-occlusive lesions including the aortoiliac and femoropopliteal arterial lesions.

CN diagnosis was made based on clinical observations such as foot deformities and direct graphy findings such as subluxation or dislocation and erosion or destruction of foot and ankle joints. Screening was done by magnetic resonance angiography before angioplasty in all patients to determine the affected BTK arteries. Interventions had been performed by antegrade approach and with the use of 5F sheaths. The ratio of balloon to vessel diameter had been planned to be 1 : 1. The balloons available during the study period had a diameter of 2.5 to 3.0 mm and a length of 60 to 120 mm. In case of flow-limiting dissection or residual stenosis of >30%, a prolonged dilation had been performed. Postintervention dual antiplatelet therapy with 100 mg aspirin and 75 mg clopidogrel once daily had been given for at least one month, and 100 mg aspirin had been given daily thereafter. Technical success was defined as restoration of direct flow in the target vessel with runoff to the foot and a residual stenosis of <30%. Once discharged, patients were followed-up in a multidisciplinary, dedicated foot clinic to facilitate the healing process and recovery of ambulatory function.

We chose 1 year prevention of amputation as the end point of this study, and amputation was defined as limb loss below or above the knee. All angiographic images and plain radiographies transferred from the radiology database were evaluated in the workstation by a radiologist with 14 years of experience. All patients were classified into two groups as diabetic patients with CLI and CN and diabetic patients with CLI only without CN.

### 2.2. Statistical Analysis

Statistical analyses were performed using Statistical Package for the Social Sciences (SPSS) 17.0 statistical software for Windows (SPSS Inc., Chicago, IL, USA). Comparisons between patients with and without Charcot neuroarthropathy were performed using the *t*-test for continuous variables and the *χ*2 test for discrete variables. Kaplan-Meier life table analysis was used to calculate limb preservation of the two groups. The log-rank test was used to compare the limb preservation rates of the groups with CN and without CN and to determine statistically significant levels. *p* values < 0.05 were considered statistically significant.

## 3. Results

63 patients met inclusion and exclusion criteria during the study period. Thus, the study consists of 63 patients with diabetic foot. Of these patients, 44 (69.8%) were men and 19 (30.2%) were women with a mean age of 67.2 years (ranging 51–84). The mean disease (diabetes mellitus) duration of all patients was 22.2 years (ranging 8–32). The most common comorbid disease was chronic renal insufficiency (34.9%). Baseline clinical characteristics were similar between the study groups. Treated lesions had a high degree of complexity in both study arms; 84.1% of the lesions were total occlusions. None of the patients in either study arms underwent inflow lesion treatment. In 12 months, the overall limb preservation rate was 81% (51/63), and the mean survival time of limb preservation after PBA was 11.07 months (std ± 0.26).

### 3.1. Diabetic Patients with CLI and CN Group

22 (34.9%) patients with diabetic foot with CN underwent conventional angiography. CN was in the right foot in 11/22; left foot, 10/22; and bilateral, 1/22. In 12 months, limb preservation was 59.1% (13/22). The mean survival time of limb salvage after PBA was 9.95 months (std ± 0.57).

### 3.2. Diabetic Patients with CLI and without CN Group

41 (65.1%) patients with diabetic foot without CN underwent conventional angiography. In 12 months, limb preservation was 92.7% (38/41). The mean survival time of limb salvage after PBA was 11.68 months (std ± 0.20).

**Figure 1:**
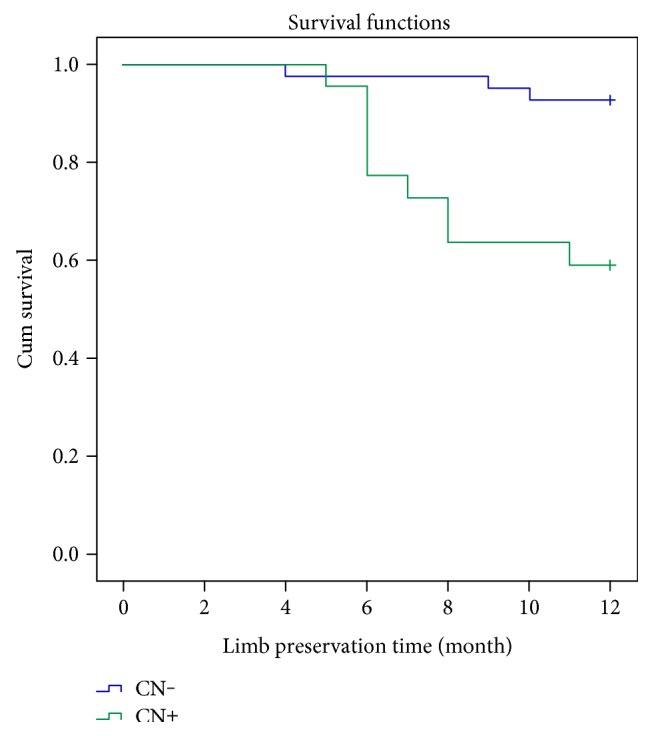
Kaplan-Meier graph comparing the limb preservation rates after endovascular treatment in diabetic patients with only CLI and in diabetic patients CLI with CN.

There was no statistically significant difference between patient age, gender, diabetic disease duration, and comorbid disease such as chronic renal insufficiency, hypertension, and coronary artery disease of the two groups (*p* > 0.05). Demographic characteristics of the patients are shown in Table 1. Limb preservation rates between CN and without CN group displayed statistically significant differences in the 12 months (Figure 1). Also, there was a statistically significant difference between the two groups in mean limb salvage time (*p* < 0.005).

## 4. Discussion

CN is a condition associated with peripheral neuropathy common in diabetic patients, characterized by joint and bone fractures, dislocation, and foot deformities [9]. It can be diagnosed clinically and radiologically, and its treatment is primarily conservative. The aim of the treatment is to create a plantigrade foot which provides bone stability [10, 11]. A stable plantigrade foot may reduce the development of foot ulcers. Developing ulcers in CN increase the risk of major amputation, and patients with diabetes and Charcot deformity associated with PAD also have a major risk of ulceration and infection [7]. CLI can be defined as a serious form of PAD that describes patients with chronic ischemic rest pain or with ischemic skin lesions, either ulcers or gangrene. Although amputation rates are not clearly known in patients with CN, patients with CN and accompanying foot ulcers have been shown to be 12 times more likely to have amputation risk than patients with CN alone [12]. Treatment of CLI in patients with CN is important in patients with impaired ulcer healing because patients with CN have a greater risk of infectious complications after surgery. Although relative ischaemia is a common contributing factor in complications of the foot in diabetes, there was not sufficient information between CN and CLI. Arterial pathology is most commonly shown to involve BTK arteries in patients with diabetes [13–16]. Revascularization in BTK arteries can be performed by surgical or endovascular methods. In recent years, endovascular revascularization, which is more comfortable, has been performed as the first choice treatment in elderly diabetic patients with lower morbidity and mortality rates, who do not require general anesthesia, especially with a high likelihood of having comorbid diseases [3, 4]. Lida et al. [17] identified diabetes as one of the factors associated with major amputation after endovascular therapy for patients with CLI due to isolated below-the-knee lesions, and they found limb preservation rate at 2 years to be 68% in patients with diabetes. Another study by Ferraresi et al. [18] found a limb preservation rate of 93% at a mean follow-up of 1048 days after infrapopliteal angioplasty in diabetic patients with CLI. Ryu et al. [19] compared clinical outcome after infrapopliteal angioplasty in CLI patients with and without diabetes and reported that the primary patency rate is lower in patients with diabetes although there was no significant difference in the limb preservation rate. Recently, Tartaglia et al. [20] found a one-year limb preservation rate of 84% after infrapopliteal angioplasty in patients at high risk of diabetes. All these studies showed the effectiveness of angioplasty on limb preservation in patients with diabetes and CLI, but there was no published study about limb preservation after angioplasty in patients with CN accompanied by CLI. In our study, the one-year limb preservation rate in all patients with ischemic diabetic foot wound who underwent endovascular treatment of BTK arteries was found to be 81%. This rate was found to be 92.7% in patients without CN and 59.1% in cases with CN. Limb preservation rates in cases without CN are similar to those in the studies in the literature. However, the lower rates in cases with CN are considered to be due to the accompanying ischemic neuropathy in these cases.

Several limitations of the present study need to be considered. Firstly, this was a retrospective study from a single institution with a small number of patients. Secondly, we did not perform follow-up angiography or use other imaging modalities for patency of the treated vascular bed. Thirdly, it is unclear whether the cause of amputation in major amputation cases was ischemia or neuropathy.

In conclusion, this study showed that diabetic CN patients may be accompanied by CLI and neuropathies may accompany ischemia in nonhealing foot ulcers. Although endovascular treatment has been shown to have higher limb preservation rates in patients who only have CLI without CN, major amputation rate is considered to be reduced with evaluation of BTK arteries and additional endovascular therapy in diabetic patients with CN. Even if surgery is planned for CN, according to us, PBA treatment before surgery is useful for limb preservation in diabetic patients with CN accompanied by CLI. However, there is a need for studies with a larger number of patients with CN.

## Figures and Tables

**Table 1:** Demographic characteristics and occlusive artery numbers of diabetic patients with Charcot neuroarthropathy and without Charcot neuroarthropathy.

	Overall patients *n*: 63	Patients with CLI and CN *n*: 22	Patients with CLI and without CN *n*: 41	
Gender (% male)	44 (69.8%)	14 (66%)	25 (61%)	*p*: 0.67
Age (years)	67.21 (std ± 8.30)	66.59 (std ± 8.87)	67.54 (std ± 8.07)	*p*: 0.59
DM disease duration (years)	22.2 (std ± 12.6)	23.4 (std ± 11.6)	21.2 (std ± 13.1)	*p*: 0.66
*Comorbid* (*n*: 54)				
Chronic renal insufficiency	24 (38%)	8 (36.3%)	16 (39%)	*p*: 0.73
Coronary artery disease	10 (15.9%)	4 (18.1%)	6 (14.6%)	
Hypertension	10 (15.9%)	3 (13.6%)	7 (17.1%)	
Patients with occlusion and stenosis	53	19 (86.3%)	34 (82.9%)	*p*: 0.65
Patients with stenosis only	10	3 (13.6%)	7 (17.1%)	*p*: 0.89

*n*: number; CN: Charcot neuroarthropathy; std: standard deviation; DM: diabetes mellitus.
